# Orbital Metastasis of Multiple Myeloma: Case Report

**DOI:** 10.4274/tjo.73645

**Published:** 2016-06-06

**Authors:** Mustafa Vatansever, Fatma Merve Bozkurt, Erdem Dinç, Eda Bengi Yılmaz, Erdinç Nayir, Ayşe Ayça Sarı, Özlem Yıldırım, Tuba Kara

**Affiliations:** 1 Mersin University Faculty of Medicine, Department of Ophthalmology, Mersin, Turkey; 2 Mersin University Faculty of Medicine, Department of Radiation Oncology, Mersin, Turkey; 3 Mersin University Faculty of Medicine, Department of Oncology, Mersin, Turkey; 4 Mersin University Faculty of Medicine, Department of Pathology, Mersin, Turkey

**Keywords:** Multiple myeloma, orbita, metastasis, plasmacytoma

## Abstract

A 68-year-old woman with a history of multiple myeloma presented to the clinic with pain and vision loss in her right eye. Proptosis was observed in her right eye and eye movements were restricted in all directions. Best corrected visual acuity was 3/10 in her right eye. On biomicroscopic examination, hyperemia and subconjunctival hemorrhage were present. Fundus examination of the right eye revealed optic disc edema and choroidal folds. In magnetic resonance imaging two orbital masses were detected. Based on the patient’s history and ocular examination, we evaluated the masses as orbital metastasis of multiple myeloma. Palliative radiotherapy was recommended.

## INTRODUCTION

Multiple myeloma (MM) is a malignancy characterized by abnormal plasma cell proliferation and is generally confined to the bone marrow. However, 3% of cases may develop extramedullary involvement, defined as the formation of solid plasmacytomas outside the bone marrow.^1^ Extramedullary involvement usually occurs in the upper skeletal system, but rarely orbital manifestations are observed.^2,3,4^ The most common ocular signs and symptoms in orbital involvement are proptosis, redness, pain, diplopia, and impaired vision; proptosis is an indicator of metastasis and recurrence. This report presents a case of orbital involvement observed during follow-up of an MM patient in remission.

## CASE REPORT

**Figure 1:**
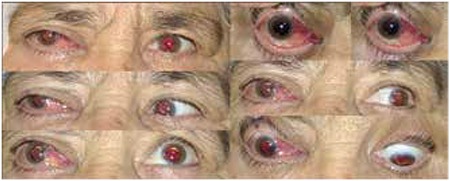
Hyperemia and subconjunctival hemorrhage were observed in the patient’s right eye and eye motility was limited in directions

**Figure 2:**
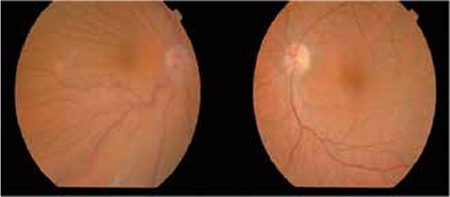
Color fundus photography revealed choroidal folds in the right eye, while the fundus appeared normal in the left eye

**Figure 3:**
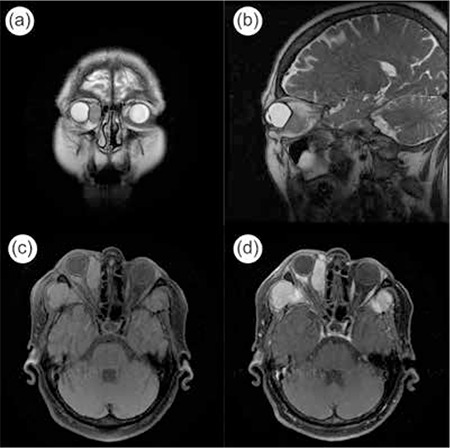
The mass located in the orbit visualized in three different planes by magnetic resonance imaging (a, b and c), and by contrast-enhanced magnetic resonance imaging (d)

**Figure 4:**
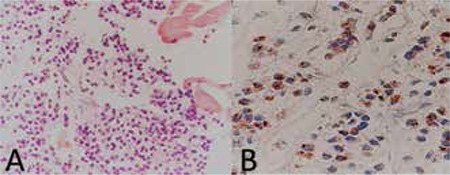
Biopsy obtained from the patient shows plasma cells in different maturation stages (A) (hematoxylin&eosin, x400) and B) Lambda light chain staining was positive (x400)

A 68-year-old female patient presented to our clinic with bilateral progressive vision loss for the previous 2 years. The patient had been diagnosed with MM 5 years earlier but was in remission at time of presentation and had no other systemic diseases in her medical history. On ophthalmologic examination her best corrected visual acuity (BCVA) was 3/10 in the right eye and 2/10 in the left eye. Anterior segment examination revealed cataract in both eyes. Posterior segment examination was normal. The patient underwent uncomplicated cataract surgery under local anesthesia on the left eye first, followed by the right eye a month later. She experienced no problems postoperatively and her uncorrected visual acuity was 7/10 in both eyes at the follow-up examination. No pathologies were observed during anterior or posterior examinations. The patient presented to our clinic about 15 days after her final follow-up appointment with complaints of pain, redness and low vision in her right eye. Her BCVA was 3/10 and 8/10 in the right and left eye, respectively. Proptosis was evident and eye movements were restricted in all directions in her right eye. On anterior segment examination of the right eye, subconjunctival hemorrhage and hyperemia were observed (Figure 1). Posterior segment examination of the right eye revealed optic disc edema and widespread choroidal folds (Figure 2). These clinical signs combined with the patient’s history of MM suggested orbital metastasis, and urgent radiologic imaging was ordered. Magnetic resonance imaging revealed two mass lesions in the right orbital space behind the globe. In contrast-enhanced images the lesions showed homogeneous enhancement, and invasion of the extraocular muscles, disruption of globe shape and optic nerve compression were observed. In addition, mass lesions showing heterogeneous enhancement were present in both temporal fossa (Figure 3). The diagnosis was confirmed using a biopsy obtained from the temporal fossa lesion (Figure 4), and the patient was referred to the radiation oncology department for consultation. Palliative radiotherapy (RT) was recommended.

## DISCUSSION

Ocular findings in MM may arise from systemic effects of the disease (increased blood viscosity) or infiltration of plasma cells into ocular tissues.^4^ These ocular findings may include crystalline corneal deposits, exudative macular detachment, ciliary body cysts and retinal hemorrhage. Though rare, orbital involvement may be an extramedullary manifestation. The most common clinical sign of orbital involvement in MM is unilateral proptosis, while hyperemia, pain, diplopia and low vision occur less often.^5^ There are also reports of bilateral proptosis in some cases.^5^ Similarly, our case presented with proptosis, subconjunctival hemorrhage, pain and vision loss. Her ocular motility limitation, optic disc edema and choroidal folds resulted from a metastatic mass located posterior to the globe which was invading the extraocular muscles and applying pressure to both the globe and the optic nerve. All of these findings have been observed in similar cases of orbital involvement.^1,2,6,7,8^

Approximately 9% of orbital tumors in adults are metastases, and orbital metastases usually originate from lung and breast cancers.^9^ There have also been reports of kidney, pancreas, prostate and gastric cancers forming orbital metastases.^10,11^ The presence of metastasis in MM indicates a poor prognosis. Orbital metastases in particular have worse survival rates compared to other extramedullary plasmacytomas.^6^ Mean expected survival for patients with recurrence is 12 months in the absence of systemic involvement, less in cases with systemic involvement. Of all malignancies occurring during remission, approximately one in three is orbital.

RT is an effective palliative therapy for MM patients, especially those with symptomatic local manifestations.^12,13^ Palliative RT is indicated for deficits related to pain, bone involvement, spinal cord compression, root compression and cranial nerve involvement. We recommended RT in this case due to the pain that accompanied the orbital involvement.

## CONCLUSION

Proptosis has an important role in the differential diagnosis of malignancies. In cases like this, imaging should be done immediately and the differential diagnosis should be considered. It should be kept in mind that proptosis can be the first sign of relapse in a patient previously diagnosed with MM.

## Ethics

Informed Consent: It was taken.

Peer-review: Externally and internally peer-reviewed.
